# Greater Cortical Thickness in Elderly Female Yoga Practitioners—A Cross-Sectional Study

**DOI:** 10.3389/fnagi.2017.00201

**Published:** 2017-06-20

**Authors:** Rui F. Afonso, Joana B. Balardin, Sara Lazar, João R. Sato, Nadja Igarashi, Danilo F. Santaella, Shirley S. Lacerda, Edson Amaro Jr., Elisa H. Kozasa

**Affiliations:** ^1^Hospital Israelita Albert EinsteinSão Paulo, Brazil; ^2^Massachusetts General Hospital–Harvard Medical SchoolBoston, MA, United States; ^3^Universidade Federal do ABCSão Bernardo, Brazil; ^4^Centro de Práticas Esportivas da Universidade de São Paulo (CEPEUSP)São Paulo, Brazil

**Keywords:** yoga, cortical thickness, aging, MRI, prefrontal cortex, meditation

## Abstract

Yoga, a mind-body activity that requires attentional engagement, has been associated with positive changes in brain structure and function, especially in areas related to awareness, attention, executive functions and memory. Normal aging, on the other hand, has also been associated with structural and functional brain changes, but these generally involve decreased cognitive functions. The aim of this cross-sectional study was to compare brain cortical thickness (CT) in elderly yoga practitioners and a group of age-matched healthy non-practitioners. We tested 21 older women who had practiced hatha yoga for at least 8 years and 21 women naive to yoga, meditation or any mind-body interventions who were matched to the first group in age, years of formal education and physical activity level. A T1-weighted MPRAGE sequence was acquired for each participant. Yoga practitioners showed significantly greater CT in a left prefrontal lobe cluster, which included portions of the lateral middle frontal gyrus, anterior superior frontal gyrus and dorsal superior frontal gyrus. We found greater CT in the left prefrontal cortex of healthy elderly women who trained yoga for a minimum of 8 years compared with women in the control group.

## Introduction

Aging is associated with changes in brain structure and function that may lead to cognitive losses, physical and behavioral changes (Persson et al., 2006; Uranga et al., 2010; Lockhart and DeCarli, 2014). Cognitive impairment in aging includes a decrease in working memory (WM; Draganski et al., 2013; Kirova et al., 2015) and in the volume of cortical areas recruited in attention tasks (Bowman and Dennis, 2015). As longevity steadily increases in the general population worldwide López-Otín et al. (2013), so does the incidence of age-related diseases, leading to higher medical costs (Grootjans-van Kampen et al., 2014). With this in mind, yoga has been considered to be a form of health-management intervention that is non-invasive as well as cost-effective (Aboagye et al., 2015).

Yoga involves the practice of postures, breathing exercises and meditation. While branches of yoga adopted in the West have a strong physical component (postures and breathing exercises), yoga is not limited to the physical body. By definition, yoga is a meditative activity embodied in physical postures in which an attentional component must be present (Taimni, 1961). Therefore, yoga is considered a contemplative practice.

Its intrinsic and unique contemplative characteristics distinguish it from typical forms of physical exercise, which are limited to the physical body. Ross and Thomas (2010) suggested that yoga has equal or greater effects on various health indicators than regular aerobic exercises. Furthermore, mind-body practices such as yoga, tai chi and meditation have been associated with positive changes in brain structure and function, especially in areas related to awareness, attention, executive functions and memory (Wei et al., 2013; Boccia et al., 2015). Recent studies reported improvement in functional connectivity (verbal, attentional and self-regulatory performance) in relation to verbal memory assessed through the Hopkins Verbal Learning Test-Revised and visuospatial memory measure, the Rey-Osterrieth Complex Test for verbal memory in older adults with mild cognition impairment who underwent yoga interventions (Eyre et al., 2016). Villemure et al. (2015) and Hernández et al. (2016) reported larger gray matter (GM) volumes in practitioners of yoga and Sahaja Yoga Meditation, respectively, compared with controls. To date, however, most studies reporting morphometric differences between mind-body intervention practitioners and non-practitioners have tested small, heterogeneous samples (Boccia et al., 2015; Tang et al., 2015). Moreover, many performed volumetric and region of interest (ROI) analyses and showed mostly mixed results, with different brain regions reported in different studies. There are even fewer studies related to the practice of yoga and morphometric differences in aging. As suggested above, it is important to investigate the effects of yoga in an increasingly older population. Thus, the aim of this study was to compare brain cortical thickness (CT) in elderly female yoga practitioners and healthy non-practitioners.

## Materials and Methods

### Participants

Twenty-one female hatha yoga practitioners who practiced at least twice a week for a minimum of 8 years, were recruited from hatha yoga studios in São Paulo, Brazil. Hatha Yoga, one of the most common yoga branches in the West, is based on asana (postures), pranayama (breathing exercise) and dhyana (meditation). We also recruited an additional group of 21 women who were naive to yoga, meditation or any mind-body intervention and were matched to the first group in age, years of formal education and level of physical activity. Subjects were matched for physical activity based on the practices of the Yoga group—those who did not practice any activity other than yoga were matched to sedentary controls and those who practiced yoga plus another physical activity were matched to a control group member who practiced the same or equivalent physical activity. Inclusion criteria were: at least 60 years of age, female, right-handed and having completed at least elementary school. We chose to include only women to add an element of homogeneity to the group. Interestingly, it was also easier to identify female yoga practitioners than male practitioners. Exclusion criteria were: substance abuse; tremor or dystonia of the head; chronic physical or other health problems that prevented them from performing their daily activities independently; any contraindication to MRI; a clinical history of neurological and/or psychiatric diseases. All volunteers provided written informed consent and the study was approved by the Institutional Review Board of Hospital Israelita Albert Einstein (CAAE 22313813.7.0000.0071).

### Questionnaires and Tests

Instrumental Activities of Daily Living—IADL: scores range from 9 (low function) to 27 (high function). Items are evaluated regarding individuals’ ability to perform each task (independently, with others’ help, or not at all; Lawton and Brody, 1969; Santos and Virtuoso, 2008).

Beck Depression Inventory—BDI: self-report questionnaire with 21 multiple choice questions addressing several depression symptoms. Scores range from 0 to 63 (Beck, 1978; Gorenstein et al., 1999).

Mini Mental State Examination—MMSE: test that evaluates several domains of cognitive function, such as spatial and temporal orientation; calculation; immediate and evoked memory; language-naming; writing; repetition and copying a drawing. Scores range from 0 to 30 (Folstein et al., 1975).

Anthropometric measurements—Weight and height were measured.

### Image Acquisition

A T1-weighted MPRAGE sequence was acquired for each participant using a Siemens 3.0T Magnetom Tim Trio System with a 12-channel head receive coil (matrix 1 × 1 × 1 mm voxel, TR = 2500 ms, TE = 3.45 ms, FOV = 265 mm, inversion time = 1100, flip angle = 7 degrees). Image quality was visually inspected immediately after each structural acquisition to control for motion effects and other artifacts.

### Cortical Thickness (CT) Analysis

The FreeSurfer analysis suite (v5.3.0 release^1^) was used to derive models of cortical surface in each T1-weighted image. These validated and fully automated procedures have been extensively described elsewhere (e.g., Dale et al., 1999; Fischl and Dale, 2000). In brief, a single filled white matter volume was generated for each hemisphere after intensity normalization, skull stripping, and image segmentation using a connected components algorithm. Then, a surface tessellation was generated for each white matter volume by fitting a deformable template. This resulted in a triangular cortical mesh for gray and white matter surfaces consisting of approximately 150,000 vertices (i.e., points) per hemisphere. Measures of CT were computed as the closest distance from the GM and white matter boundary to the GM and cerebrospinal fluid boundary at each vertex on the tesselated surface. Mean CT across the entire brain was also computed for each participant. Thickness data were smoothed using a 15-mm surface-based smoothing kernel.

### Statistical Analyses of the Images

Whole-brain CT analysis was then performed on each vertex using the general linear model (GLM) embedded in the SurfStat toolbox^2^ for Matlab (R2010b; MathWorks). Participants’ age was included as a covariate to control for the effect of age on brain structure. CT was compared in yoga practitioners vs. non-practitioners. We also examined different age effects on CT between groups by conducting a group by age interaction. Corrections for multiple comparisons across the whole brain were performed using random-field theory (RFT) cluster-extent based thresholding for nonisotropic images (Worsley et al., 1999). Group differences in mean CT were assessed by an independent samples *t*-test.

### Questionnaires and Anthropometric Statistical Analysis

Data obtained from questionnaires and anthropometric measurements were analyzed using the SPSS 17.0 program (SPSS Inc., Chicago, IL, USA). Variables were compared using Student’s *t* or Mann Whitney tests.

## Results

The yoga group had 14.9 years of hatha yoga practice, on average. There were no significant differences between groups in terms of age, years of education, questionnaire scores or anthropometrical measures, as shown in Table 1.

**Table 1:** Group characteristics.

	Control Group (*n* = 21)	Yoga Group (*n* = 21)	*p*
Age (years)	67.9 (1.004)	66.2 (0.98)	0.24
Years of Education	14.6 (0.42)	14.1 (0.42)	0.35
BMI, kg/m^2^	25.3 (0.63)	24.5 (0.92)	0.53
BDI	7.4 (1.2)	5.3 (0.98)	0.18
MMSE	28.8 (0.28)	28.1 (0.38)	0.16
IADL	26.8 (0.14)	26.9 (0.04)	0.14
Years of yoga practice	0.0	14.9 (1.77)	

*Data expressed as mean (± standard error); BMI, body mass index; BDI, Beck Depression Inventory; MMSE, Mini Mental State Examination; IADL, Instrumental Activities of Daily Living*.

### Between-Group Differences in Cortical Thickness

**Figure 1:**
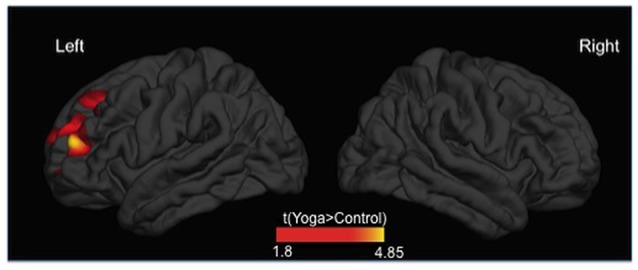
Differences in cortical thickness (CT) between yoginis and controls (*P* < 0.05, cluster corrected). Yoga practitioners showed greater CT in a cluster in the left prefrontal cortex (RTF-based, cluster-corrected, *p* < 0.05).

Relative to controls, yoga practitioners (i.e., female yoga practitioners) showed a significantly greater CT in a left prefrontal lobe cluster (cluster forming threshold *p* < 0.05, cluster corrected *p*-value = 0.01574), which included anterior and lateral portions of middle and superior frontal gyri (BA8/9; Figure 1, Table 2). No regions exhibited decreased CT in yoginis compared to controls. The age by group interaction was also not significant. There were no significant between-group differences in mean CT for the whole cortex, as assessed for each hemisphere individually (*t*_(40)_ = −0.690, *p* = 0.494).

**Table 2:** Anatomical and statistical information of the cluster in which significant between-group differences in cortical thickness (CT; i.e., yoga practitioners > Controls) were detected.

Region	Side	Talairach/MNI Coordinates			Peak vertex *t*-value*
		*X*	*Y*	*Z*	
Middle frontal gyrus	L	−35/−35	47/49	10/10	4.85
Superior frontal gyrus	L	−20/−20	54/57	15/17	3.06

*Local maxima within the cluster.

## Discussion

In the present cross-sectional study, we observed greater CT in the left prefrontal cortex (middle and superior frontal gyri) of healthy elderly women who trained yoga for a minimum of 8 years compared with women in the control group. The present results parallel those previously reported in which younger yoga and meditation practitioners had greater GM volumes than non-practitioners compared to non-practitioners in the following brain regions: larger GM volume in the right anterior insula and right inferior temporal gyrus (Hernández et al., 2016), increased GM volume in the frontal lobe (right anterior cingulate cortex and left middle and medial frontal gyrus), left precuneus and fusiform gyrus and right thalamus (Boccia et al., 2015), increased GM volume in the left mid insula, left frontal operculum, left orbitofrontal cortex, right middle temporal gyrus and right primary somatosensory cortex/superior parietal lobule (Villemure et al., 2015). Our sample consisted of healthy elderly Brazilian women with 14.3 years of education, on average (which is high compared to the national average of 4.7 years among people over 60 years of age; IBGE—Instituto Brasileiro de Geografia e Estatística, 2014). The volunteers in this study did not present any severe cognitive impairments, as assessed by the MMSE, nor any problems associated with functionality in daily life activities (as assessed with the IADL). Importantly, no participants displayed any depressive symptoms, as these may be associated with cognitive alterations (McClintock et al., 2010; Dong et al., 2016), or with an early manifestation of dementia (Panza et al., 2010). Therefore, it is unlikely that the differences observed between groups are due to demographic characteristics or depressive symptoms.

To the best of our knowledge, this is the first imaging study showing greater CT in elderly female yoga practitioners relative to controls. The greater thickness was observed in left prefrontal lobe areas associated with attention and other executive functions (Jeon, 2014). When performing a yoga posture, muscles are engaged for a minimum amount of time in a state of attention (processed in the prefrontal cortex, PFC), similarly to what occurs in meditation (Kane and Engle, 2002; Koechlin et al., 2003). The meditative process (for which attention is essential) is associated with increased oxyhemoglobin concentrations in the PFC due to the increased blood flow to that region (Deepeshwar et al., 2015; Singh et al., 2016). Similarly, alterations in attention and cognition result in different degrees of electrical activity in the PFC (Aftanas and Golocheikine, 2001; Davidson et al., 2003; Lutz et al., 2004; Desai et al., 2015). In our study, we observed alterations in areas associated with executive functions of attentional control rather than motor regions.

Other attentional tasks such as video game playing, cause changes in cognition in older adults (Toril et al., 2014) and brain structure of adolescents, with positive correlation between CT and hour per week of video game in the left dorsolateral prefrontal cortex in left middle frontal gyrus and left frontal eye fields (Kühn et al., 2014). Playing video game may lead to addiction and its deleterious effects, however, yoga is associated with improvement in mental health, therefore different psychobiological mechanisms should be involved in these trainings (Cramer et al., 2013; Pascoe and Bauer, 2015).

Attention-Deficit/Hyperactivity Disorder (ADHD) is related to differences in brain structure and function when compared to controls (Batty et al., 2015; Silk et al., 2016). Yang et al. (2015) and showed thinner superior frontal gyrus in the right hemisphere in children with ADHD compared to healthy controls. Hoekzema et al. (2012) observed reduced laminar CT in a bilateral area from the superior frontal gyrus to the precentral gyrus, inferior and superior parietal cortex. Some of these morphometric abnormalities in ADHD are associated to attention and WM. Furthermore, du Boisgueheneuc et al. (2006) observed that WM was compromised in patients with lesions in the left superior frontal gyrus, one of the regions where we found greater CT in our study. WM allows temporary storage of data and directs attention to relevant information (Baddeley, 1992; Cowan, 2008). It is, therefore, an essential resource of meditative processes, where the object of attention (i.e., breathing) becomes the meaningful content. Moreover, WM capacity decreases with age (Sander et al., 2012). Thus, while aging is associated with a decrease in GM volume in the frontal lobes (Tisserand and Jolles, 2003; Salat et al., 2004; Lockhart and DeCarli, 2014) along with WM deterioration, the practice of yoga appears to have neuroprotective effects, thus having a positive impact on mental health among the elderly. We believe that yoga may preserve cognition, which is supported by our findings as well as previous results in which younger yoga and meditation practitioners showed sharper cognitive functions relative to controls. Eyre et al. (2016) showed that older adults with mild cognitive impairment who practiced 12 weeks of yoga had improvement in functional connectivity (verbal, attentional, and self-regulatory performance) in relation to verbal memory assessed through the Hopkins Verbal Learning Test–Revised and a visuospatial memory measure, the Rey-Osterrieth Complex Test. Considering Taichi chuan a mind-body activity, therefore a mental training, similar to yoga, there were differences in the cortex performing vertex-wise analyses. Wei et al. (2013) reported thicker cortex in Taichi chuan practitioners compared to control group: medial occipitotemporal sulcus, superior temporal gyrus and lingual sulcus in left hemisphere and the circular sulcus of the insula, precentral gyrus and middle frontal sulcus in right hemisphere. Furthermore, Gard et al. (2014) showed that meditation may reduce cognitive decline associated with normal aging, and Sharma et al. (2014) observed greater enhancement in cognitive domains such as memory retention and attention in yoga practitioners relative to controls. Gothe et al. (2014) found that yoga practitioners had greater WM capacity compared with a control group that did stretching and strengthening exercises. In that study, the different results may be due to the attention/awareness component found in yoga but not in stretching and strengthening. Chandra et al. (2016) also reported greater WM capacity in Sudarshan Kriya yoga practitioners. Even though the studies mentioned above detected cognitive alterations, they did not perform imaging tests.

Our study has some limitations. Ideally, individuals across groups should have performed the same physical activities (other than yoga). Matching participants on this variable proved to be very difficult. Thus, we matched as best we could based on relative physical effort. Furthermore, as the number of participants in each group was relatively small, it did not allow us to make comparisons between individuals from different ethnic groups. Also, our volunteers performed only one MRI scan. Future studies should involve longitudinal randomized controlled trials and correlations with other peripheral measures.

In conclusion, healthy elderly women who practiced hatha yoga for at least 8 years had greater prefrontal CT than a group of matched controls. This CT may be associated with cognitive preservation.

## Author Contributions

RFA: acquisition of data; design; interpretation; revising and final approval of the article; agreement to be accountable for all aspects of the work. JBB and JRS: analysis; revising and final approval of the article; agreement to be accountable for all aspects of the work. SL and SSL: interpretation; revising and final approval of the article; agreement to be accountable for all aspects of the work. NI: acquisition of data; final approval of the article; agreement to be accountable for all aspects of the work. DFS: interpretation; final approval of the article; agreement to be accountable for all aspects of the work. EA: revising and final approval of the article; agreement to be accountable for all aspects of the work. EHK: conception and design of the work; analysis and interpretation of data; revising and final approval of the article; agreement to be accountable for all aspects of the work.

## Conflict of Interest Statement

The authors declare that the research was conducted in the absence of any commercial or financial relationships that could be construed as a potential conflict of interest.

## Footnotes

^1^http://surfer.nmr.mgh.harvard.edu/

^2^http://www.math.mcgill.ca/keith/surfstat/
